# Buffalo Medical Association. Address of the President, Josiah Trowbridge, M.D.

**Published:** 1845-09-01

**Authors:** 


### EDITORIAL, MEDICAL INTELLIGENCE, &c.

Buffalo Medical Association. *Address of the President, Josiah Trowbridge, M. D.*—The first regular meeting of the Association was on the 5th ultimo. The majority of the physicians of the city were present, and signified their intention to unite in fulfilling its objects. A case of ossification of the aortic valves, with the morbid specimen, was presented, which led to the discussion of various topics connected with cardiac affections. A committee was appointed to collect statistics of fractures, with reference to the proportion of cases in which a perfect restorement of the limb is effected. We shall speak of this subject hereafter.

On the opening of the meeting, the President Dr. Josiah Trowbridge addressed the association at some length. Dr. T. has kindly acceded to our request to be allowed to present portions of his remarks to our readers. We regret that our restricted limits oblige us to curtail any of their fair proportions.

After some preliminary remarks which we omit, the President proceeded to speak of the purposes of the association.

The objects which we propose to accomplish, as set forth in the preamble to the constitution, are as follows. First, a free and mutual interchange of Medical opinions. This part of the preamble is intended, undoubtedly, to embrace verbal and written communications ; reports of cases ; minutes of autopsical examinations ; in fact, any thing which may be deemed new, extraordinary, or interesting to the profession. This kind of intercourse, at all times advantageous, will be peculiarly so whenever an epidemic of a malignant character prevails. Most of us, or, at least, the elder members of the profession, recollect with pain and mortification the different views taken by physicians, of the proper modes of treating the several Epidemic diseases which have at different periods visited our country, on their first appearance. On such occasions the public becomes excited and alarmed. Members of the faculty not agreed among themselves, each confident in the course which *he* thinks most successful or most likely to be so ; and, probably, not contented with carrying out their own individual views, are too apt to reflect illiberally, severely perhaps, upon the practice of others. Is it strange that under such circumstances the public should lose confidence in the profession ; or that they should seek for aid among the hosts of charlatans and quacks, who always infest every community; and who, at least, agree on one point, that they can certainly and invariably cure! It is under such circumstances that an association like this, becomes eminently important and useful, both to ourselves and to the public.

Secondly—To cultivate a friendly intercourse, an honorable and gentlemanly deportment, and a strict observance of medical courtesy towards each other. This portion of the preamble embraces a wide domain. It applies to private as well as to public and professional intercourse. It includes the whole code of medical ethics, whether expressed or understood, as well as moral standing and character. The usefulness, the harmony, and the perpetuity of this association, depend upon a rigid adherence to, and an honest determination, on the part of its members, to carry out in its broadest sense the spirit, as well as the letter of this portion of the preamble. * * * * It is agreed on all hands that quackery out of the profession is one of the greatest evils of the day, in my estimation quackery in the profession is worse.

After dwelling at some length on the subject of medical ethics, and specifying divers violations thereof by way of illustrations, the Doctor proceeded to speak of the present condition of the profession and the means of improvement. We quote his views on this very important topic.

It is, I believe, generally admitted by the faculty, and, as generally lamented, that the profession is retrograding in professional attainment, in respectability, and in public estimation. This remark is intended, of course to be general ; that there are numerous and honorable exceptions is most certainly true. If such be the fact, it is well worth while to enquire into the causes. This subject has attracted the attention of medical men throughout the state, and if this *fact* has not been generally admitted, all agree that it is important that some measures be adopted to raise the standard of medical attainment. The measures heretofore pursued to effect the object have been legislative enactments. Unfortunately this method has failed to accomplish the object. The ignorant, the designing and the interested, by misrepresentation and falsehood have succeeded to a great extent in exciting public prejudice. They have represented that we are monopolists, a protected and an aristocratic body; that our system could not be sustained unless supported by legislative enactments, and that these enactments are anti-republican and contrary to the free spirit of our institutions. These prejudices have not been confined to the vulgar and uneducated, but have extended to the halls of our legislature; applications to that body for enactments for the protection, and solely for the benefit of the public, have been refused; our motives misrepresented and misconstrued; the value of medical attainment derided; and finally, by a deliberate and solemn act of legislation, they have in so far as was in their power, degraded the medical profession to the level of the Homeopathist and Steam Doctor. As an individual of the profession, I am, therefore, opposed to any further applications to the legislature; and I am not sure, indeed, I am rather inclined to the opinion, that it would be better if every law regulating the practice of physic and surgery were repealed. This however, I am inclined to believe, is not the opinion of the faculty generally; the balance of opinion I should think was in favor of maintaining, or rather submitting, to the laws as they now stand. All however, seem to be of the opinion that alteration is desirable, but without agreement as to the mode. It appears to me that it remains with the profession, collectively and individually, and without the aid of legislative enactments, to correct the evils which are acknowledged to exist.

By an united and proper effort much may undoubtedly be accomplished. It has always struck me that the great evil, and one that lies at the foundation of all the rest, is the non-requirement of a good classical education in a student, previous to his being permitted to commence his medical studies. If the state medical society would pass resolutions, recommending to, and enforcing upon the several Medical Colleges, together with the state and county medical societies, not to licence or grant diplomas unless the applicant was possessed of a competent knowledge of the greek and latin languages, mathematics, and natural philosophy; it seems to me that this would go far to cure the evils which are acknowledged to exist. Physicians, also, should be requested not to admit an applicant to enter their offices unless he is possessed of these prerequisites.

In connexion with this part of the subject, I would beg leave to remark, that a custom has become prevalent among the profession in this city, which I esteem very objectionable, viz: allowing students a compensation for the privilege of entering our offices—in fact offering a bounty to unqualified young men to enter upon the study of the profession, as well as imposing an onerous tax upon ourselves. This evil, it seems to me, might be corrected by an understanding among the members of this society that no student be permitted to enter our offices without paying a moderate fee for tuition.

If then the position be correct, that it depends upon the faculty, individually and collectively, to accomplish these important objects, shall we not one and all cordially unite our efforts for their attainment? If I am correctly informed, the faculty of this city have hitherto enjoyed an enviable reputation for their urbanity and gentlemanly deportment towards each other, and for integrity and fairness in their medical intercourse. I doubt not, that it will be the highest ambition of every member of this association, not only to maintain the reputation we have acquired, but to elevate it still higher.

It is unnecessary to commend to the attention of our readers the above, as the reflecions of one who unites with great practical experience and eminence as a practitioner, an ardent zeal for the elevation of our profession, and who is distinguished as exemplifying those principles of integrity, honor and courtesy, upon which the dignity, and, in a great degree, the usefulness of the profession depends.

The unanimity and spirit exhibited at the first meeting of the Association authorise the expectation that the objects for which it has been instituted will be accomplished.

☞ Our city readers will recollect that the next meeting is on Tuesday, the 2d inst., at the office of Dr. James P. White. By a resolution of the Association, non-resident physicians of regular standing, are cordially invited to attend the meetings.

